# Supplementary figures and images for: Venous malformation vessels are improperly specified and hyperproliferative
Source: PLoS One. 2021 May 27;16(5):e0252342. doi: 10.1371/journal.pone.0252342 (PMC8158993; doi:10.1371/journal.pone.0252342)

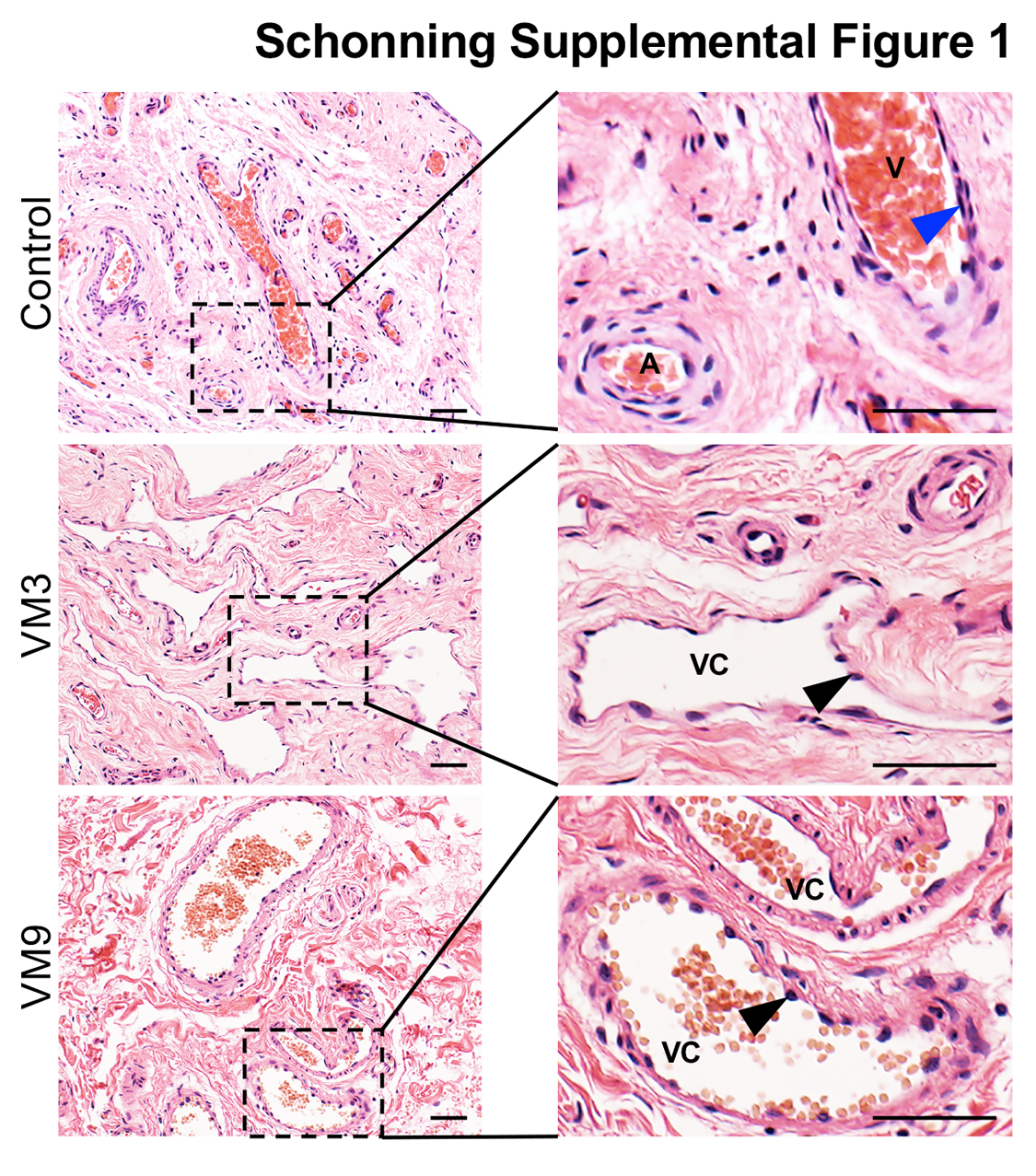

Supplement: S1 Fig — Representative H&Es of VMs and control neonatal skin. Boxed areas are enlarged to the right. Blue arrowheads highlight normal EC morphology. Black arrowheads mark ECs with abnormal morphology. A-artery, V-vein, VC-VM channel. Scale bars—50μm. (TIF) [file pone.0252342.s001.tif]

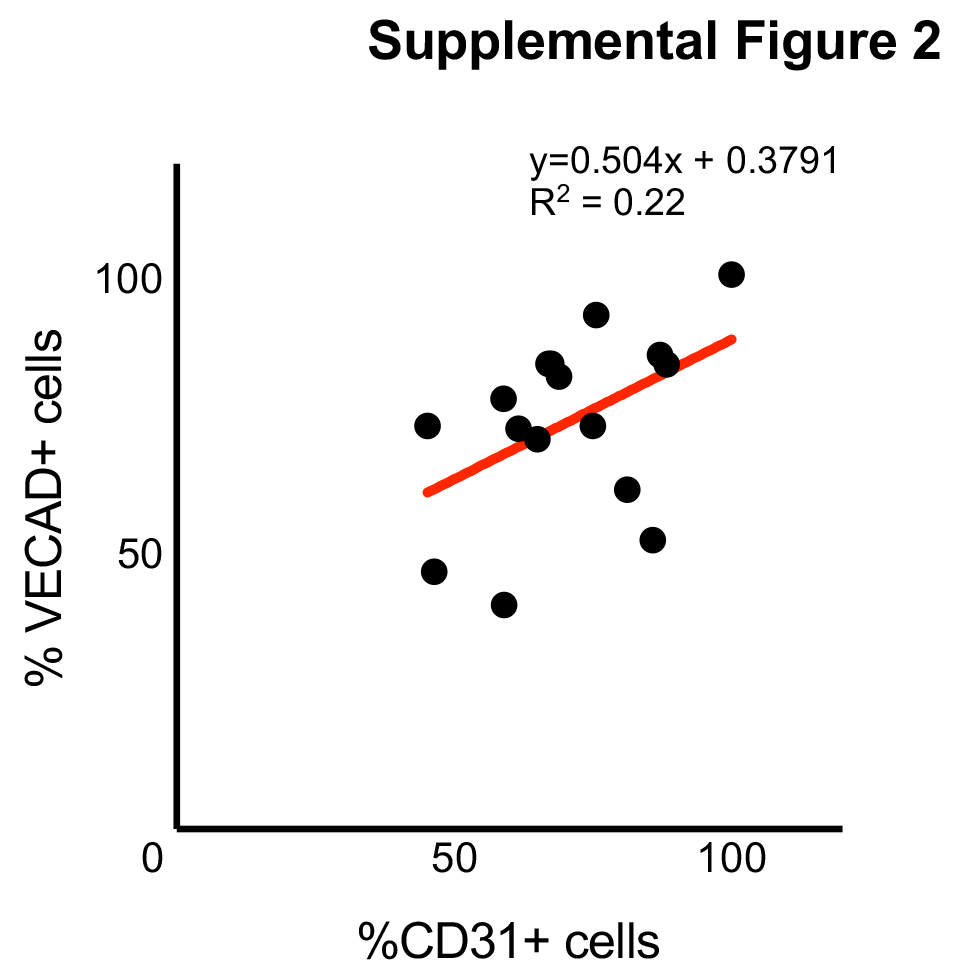

Supplement: S2 Fig — A) Representative sections of VMs and control neonatal dermis co-stained for CD31 and PDGFRβ. Red open arrowheads mark CD31+/PDGFRβ- ECs, yellow arrowheads mark CD31-/PDGFR β+ mural cells. White arrowheads mark CD31+/PDGFR β+ cells. A, B) Boxed areas are enlarged to the right. V-vein, VC-VM channel. Scale bars—50μm. V-vein, VC-VM channel. (TIF) [file pone.0252342.s002.tif]

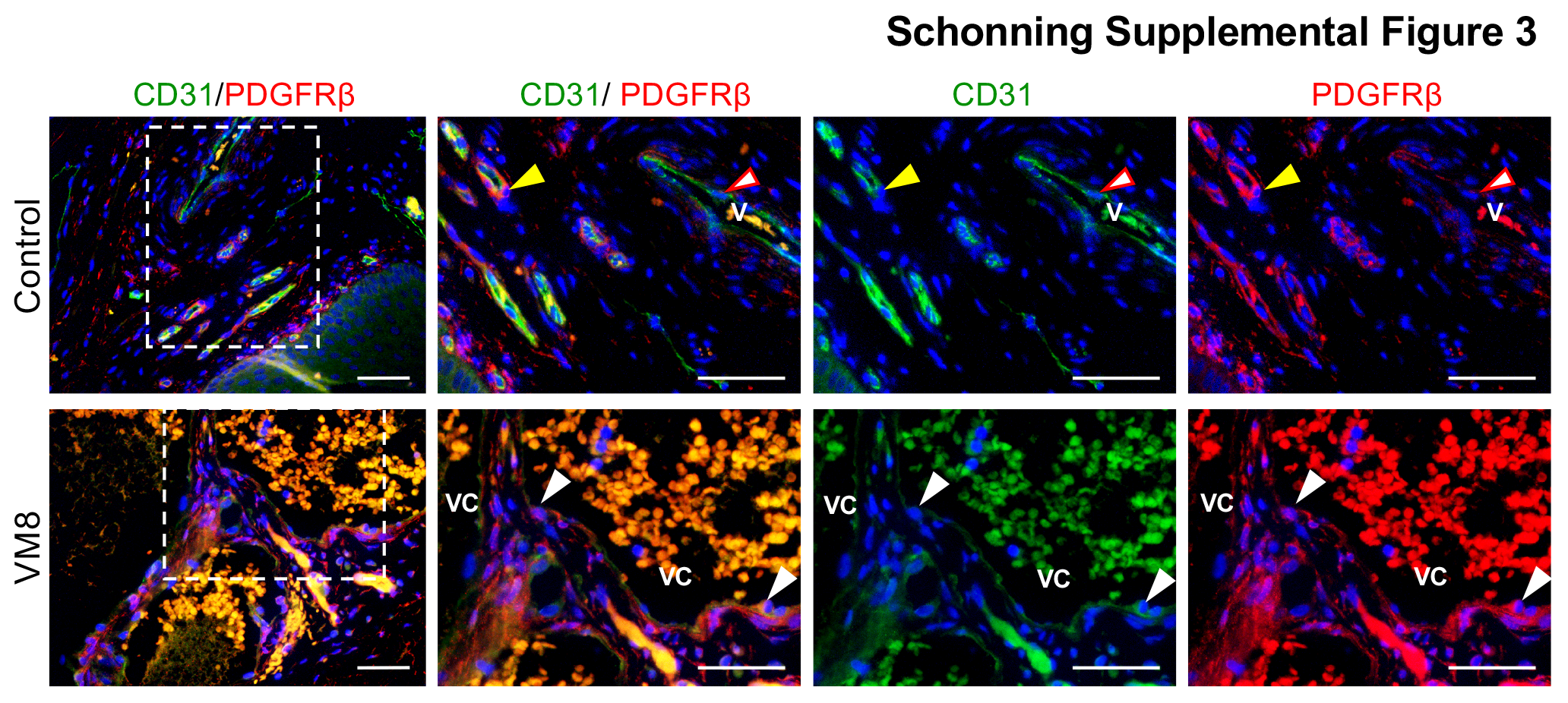

Supplement: S3 Fig — Neonatal dermis stained for NOTCH3 (left) and neonatal dermis stained for NOTCH3 which was blocked by pretreatment with a NOTCH3 blocking peptide (right). A-artery, V-vein. Scale bar—50μm. (TIF) [file pone.0252342.s003.tif]

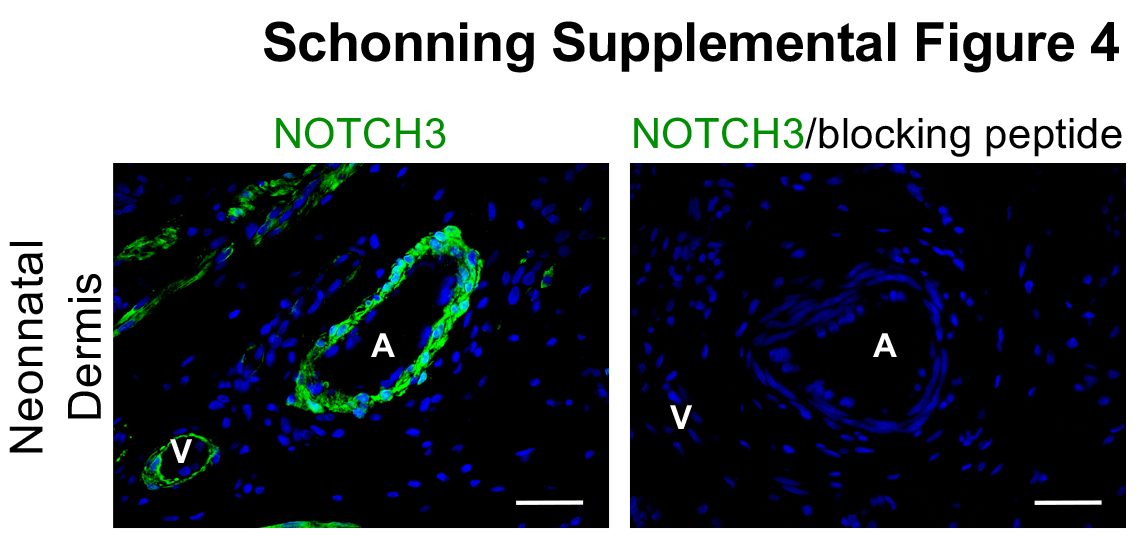

Supplement: S4 Fig — A fetal tissue array was co-stained for NOTCH3 and CD31. Representative images from adrenal gland, gallbladder, and umbilical cord shown. Boxed areas are enlarged to the right. Yellow arrowheads mark VECADHERIN-/NOTCH3+ mural cells, and white arrowheads mark VECADHERIN+/NOTCH3+ ECs. Scale bars—50μm. (TIF) [file pone.0252342.s004.tif]

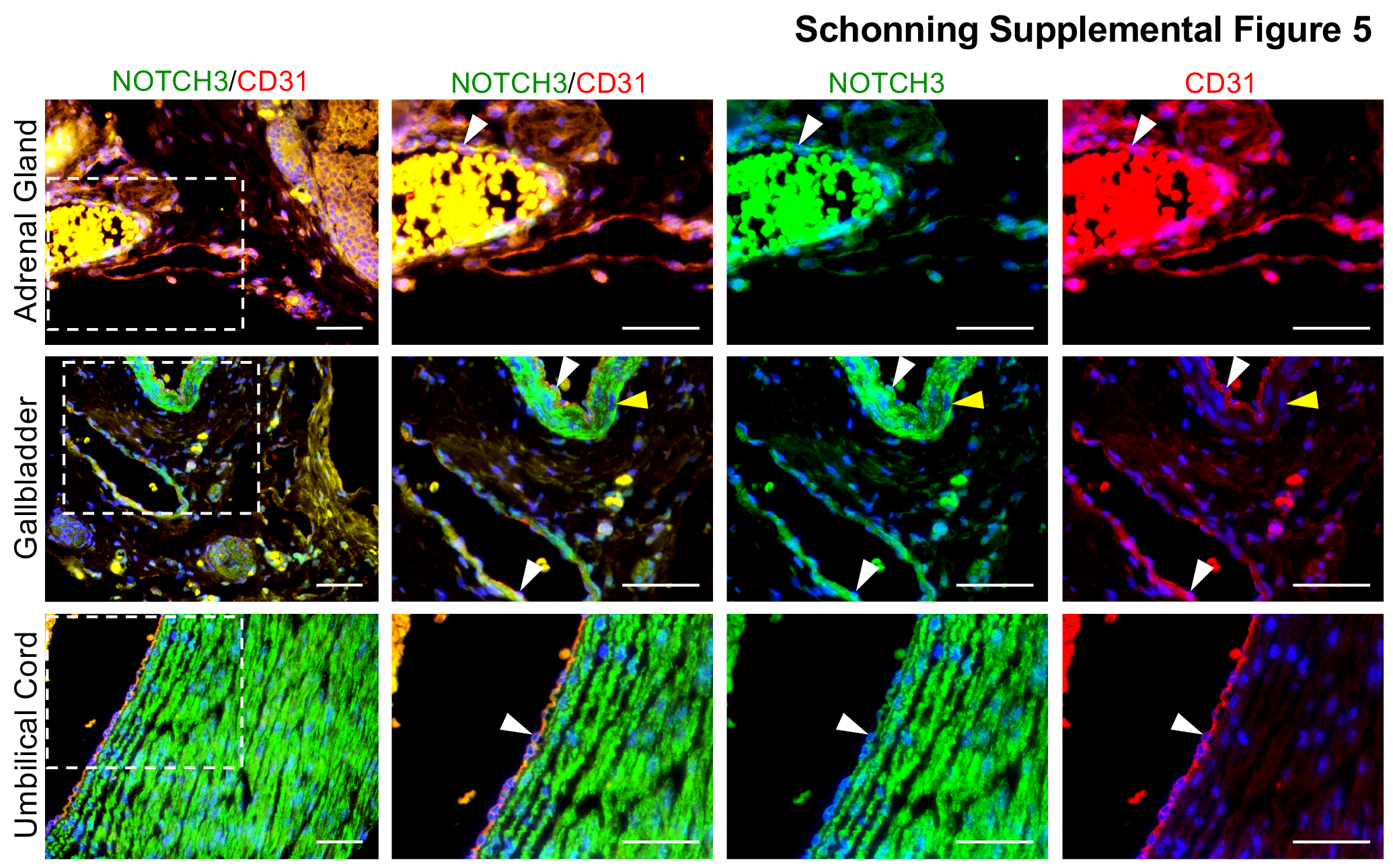

Supplement: S5 Fig — A) Representative sections of VMs and control neonatal dermis co-stained for VECADHERIN and CD146. White arrowheads mark VECADHERIN+/CD146+ cells. A, B) Boxed areas are enlarged to the right. V-vein, VC-VM channel. Scale bars—50μm. V-vein, VC-VM channel. (TIF) [file pone.0252342.s005.tif]

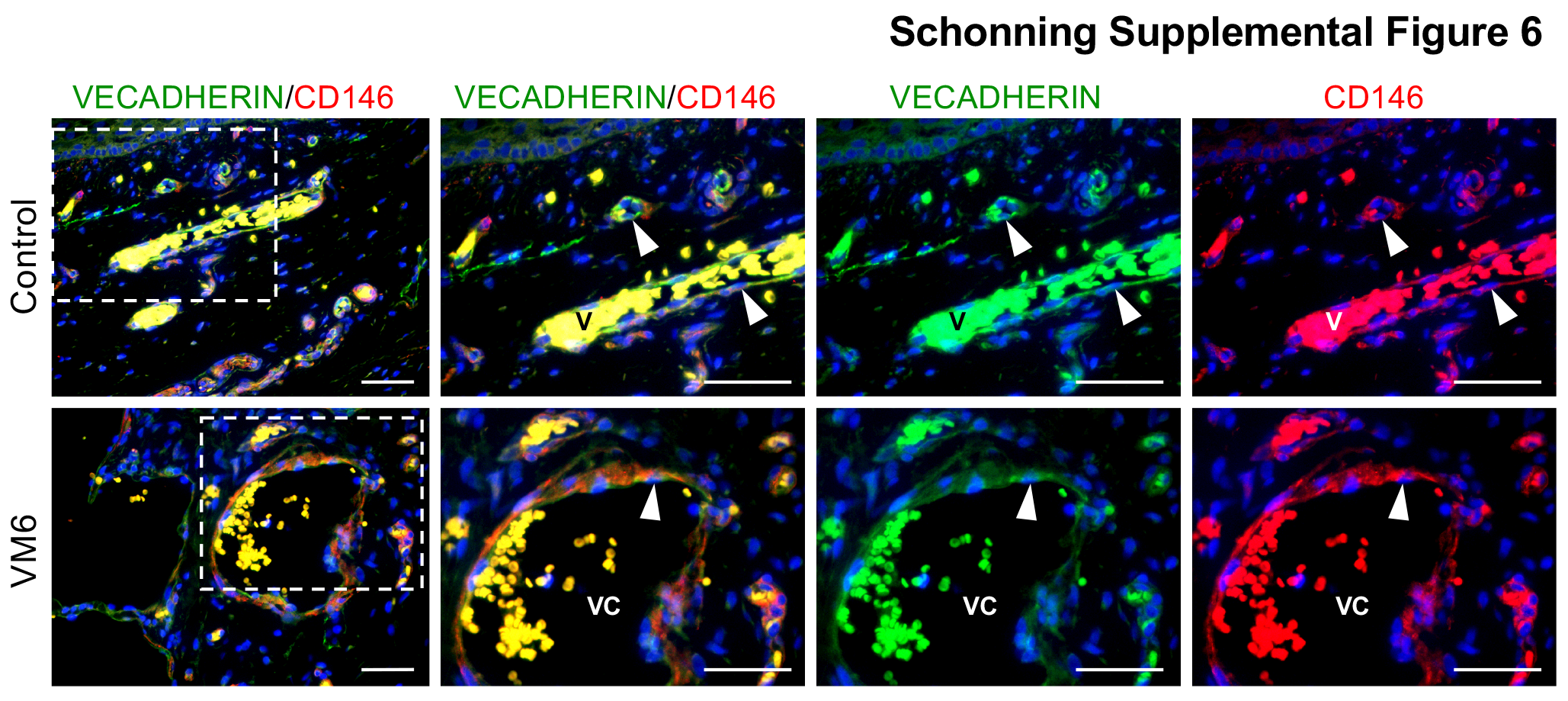

Supplement: S6 Fig — (TIF) [file pone.0252342.s006.tif]
