# Supplementary material for: Venous malformation vessels are improperly specified and hyperproliferative
Source: PLoS One. 2021 May 27;16(5):e0252342. doi: 10.1371/journal.pone.0252342 (PMC8158993; doi:10.1371/journal.pone.0252342)
Supplement: S1 Table — (DOCX) [file pone.0252342.s007.docx]

| **S1 Table. List of antibodies, sources, and dilutions** | | | |
| --- | --- | --- | --- |
| **Antigen** | **Vendor** | **Catalog #** | **Dilution** |
| CD31 | DAKO | MO823 | 1:100 |
| VECADHERIN | RnD Systems | AF938 | 1:100 |
| VEGFR2 | RnD Systems | AF357 | 1:100 |
| COUP-TFII | RnD Systems | PP-H7147-00 | 1:100 |
| EPHB4 | RnD Systems | AF446 | 1:100 |
| EPHRINB2 | RnD Systems | AF496 | 1:100 |
| DLL4 | RnD Systems | AF1389 | 1:100 |
| PDGFRβ | Abcam | ab32570 | 1:100 |
| αSMA-Cy3 | Sigma | C6198 | 1:1000 |
| NOTCH3 | Abcam | ab23426 | 1:500 |
| NOTCH3** | Abcam | ab60087 | 1:100 |
| CD133 | Millipore | MAB4399 | 1:50 |
| CKIT | Abcam | ab5505 | 1:100 |
| CD146 | Abcam | ab7569 | 1:100 |
| KI67 | Abcam | ab15580 | 1:100 |
| *antibody used for fetal array | | | |
