# Supplementary material for: Venous malformation vessels are improperly specified and hyperproliferative
Source: PLoS One. 2021 May 27;16(5):e0252342. doi: 10.1371/journal.pone.0252342 (PMC8158993; doi:10.1371/journal.pone.0252342)
Supplement: S2 Table — (DOCX) [file pone.0252342.s008.docx]

| **S2 Table. Summary of NOTCH3 expression in ECs of fetal organs** | |
| --- | --- |
| **Organ** | **NOTCH3+ endothelium (%)** |
| Adrenal Gland | 100% |
| Bone | 20% |
| Brain | ND* |
| Colon | 100% |
| Epididymus | 100% |
| Eye | 50% |
| Fallopian Tubes | ND* |
| Gallbladder | 100% |
| Heart | 50% |
| Kidney | 0% |
| Liver | 0% |
| Lung | 50% |
| Muscle | 0% |
| Ovary | ND* |
| Placenta (maternal & fetal) | 0% |
| Rectum | 100% |
| Skin | 100% |
| Small Intestine | 100% |
| Smooth Muscle | 100% |
| Spleen | ND* |
| Stomach | 100% |
| Thymus | ND* |
| Umbilical Cord | 100% |
| Uterus (fetal) | 0% |

*ND, not determined due to poor tissue quality
